# Chitosan–Glycerol Injectable Hydrogel for Intratumoral Delivery of Macromolecules

**DOI:** 10.3390/gels11080607

**Published:** 2025-08-02

**Authors:** Robert L. Kobrin, Siena M. Mantooth, Abigail L. Mulry, Desmond J. Zaharoff, David A. Zaharoff

**Affiliations:** 1 Lampe Joint Department of Biomedical Engineering, University of North Carolina-Chapel Hill and North Carolina State University, Raleigh, NC 27695, USA; rlkobrin@ncsu.edu (R.L.K.);; 2 UNC Lineberger Comprehensive Cancer Center, Chapel Hill, NC 27599, USA; 3 NC State Comparative Medicines Institute, Raleigh, NC 27606, USA

**Keywords:** hydrogel, injectable, chitosan, glycerol, intratumoral, delivery

## Abstract

Intratumoral injections of macromolecules, such as biologics and immunotherapeutics, show promise in overcoming dose-limiting side effects associated with systemic injections and improve treatment efficacy. However, the retention of injectates in the tumor microenvironment is a major underappreciated challenge. High interstitial pressures and dense tumor architectures create shear forces that rapidly expel low-viscosity solutions post-injection. Injectable hydrogels may address these concerns by providing a viscoelastic delivery vehicle that shields loaded therapies from rapid expulsion from the tumor. A chitosan–glycerol hydrogel was thus developed and characterized with the goal of improving the injection retention of loaded therapeutics. The gelation parameters and mechanical properties of the hydrogel were explored to reveal a shear-thinning gel that is injectable through a 27-gauge needle. Biocompatibility studies demonstrated that the chitosan–glycerol hydrogel was nontoxic. Retention studies revealed significant improvements in the retention of model therapeutics when formulated with the chitosan–glycerol hydrogel compared to less-viscous solutions. Finally, release studies showed that there was a sustained release of model therapeutics of various molecular sizes from the hydrogel. Overall, the chitosan–glycerol hydrogel demonstrated injectability, enhanced retention, biocompatibility, and sustained release of macromolecules, indicating its potential for future clinical use in intratumoral macromolecule delivery.

## 1. Introduction

Immunotherapy has revolutionized cancer treatment over the past decade. However, current cancer immunotherapies are dose-limited and cause significant adverse events due to their systemic administration. Between 70 and 90% of patients receiving intravenous (i.v.) injections of immune checkpoint inhibitors (ICIs), the dominant class of immunotherapy, suffer from at least one immune-related adverse event (irAE), with grade 3+ irAEs typically occurring in 20–50% of patients [1,2]. Due to these toxicities, lower doses of immunotherapies must be used. At these lower doses, systemically administered immunotherapies, be they ICIs, bispecific antibodies, or immune-stimulating cytokines, such as interleukin-2 (IL-2) or interferon-alpha (IFN-α), are unlikely to reach optimal concentrations within a tumor [3].

Direct injection of immunotherapy into one or more tumors may help maximize local concentrations of immunotherapeutics while minimizing systemic toxicities. Recent studies have shown that intratumoral (i.t.) injections result in maximum concentrations (Cmax) that are several orders of magnitude higher in tumors and have higher efficacies compared to i.v. or s.c. injections [3,4]. Clinical interest in i.t. delivery has increased in recent decades, and modern image-guided injection techniques now make it possible to inject most, if not all, solid tumors. Currently, the oncolytic herpes virus talimogene laherparepvec (T-VEC), an advanced melanoma treatment, is the only immunotherapy approved for i.t. delivery [5]. However, just in the last 5 years, there have been more than 400 clinical trials evaluating i.t. immunotherapies (www.clinicaltrials.gov key search terms: “intratumoral” OR “intralesional” AND “cancer” AND “immunotherapy”).

Despite this promising trend, a major underappreciated barrier to the success of i.t. immunotherapy is the rapidity with which macromolecule therapeutics drain from an injected tumor. The i.t. residence times of direct injected cytokines and monoclonal antibodies are brief—typically 8–24 h [6,7,8]. Short residence times limit antitumor efficacy and necessitate frequent, repeated injections, which are impractical [9].

The deposition and retention of i.t. injected therapeutics are limited by physical barriers within the tumor microenvironment. Dense tumor architectures and high interstitial pressures rapidly exclude saline-based i.t. injections via the needle track [10,11]. Furthermore, intense pressures at the needle tip result in unpredictable tumor cracking and leakage [12]. Overall, a significant fraction, if not a majority, of i.t. injected solutions leak from tumors into lower pressure surrounding tissues.

Formulating immunotherapies within a local delivery and retention system can overcome these challenges. Hydrogels, which are viscoelastic solids composed of three-dimensional networks of crosslinked polymers, have been explored to help localize a variety of therapeutics [13]. The porous nature of hydrogels, which are typically >90% water, allows for facile loading and the sustained release of macromolecules in a controlled, sustained manner through either natural diffusion or swelling [14]. Hydrogels comprising natural biopolymers and formulated in aqueous conditions, without organic solvents, offer the greatest opportunity for localized delivery of labile immunotherapeutics. Furthermore, the varying pore sizes of different hydrogels allow for the delivery of both small immunotherapeutics, such as peptides and agonists, as well as large immunotherapeutics, such as cytokines, antibodies, and cells [15,16,17]. Other useful hydrogel qualities for localized delivery include tunable mechanical properties, tunable release kinetics, and diverse chemical properties through the use of various polymers. The myriad advantages of hydrogel-based delivery systems have been recently reviewed [18,19,20,21,22,23,24].

This project investigates the potential of an injectable chitosan–glycerol hydrogel for local delivery and retention of immunotherapeutics. Chitosan is a biodegradable, cationic polysaccharide derived from chitin [25]. It is primarily harvested from the exoskeletons of crustaceans; however, non-animal sources are becoming more prevalent. Chitosan is a component of FDA-approved wound dressings and is widely used in preclinical delivery systems. It is commonly used in hydrogel applications due to its biocompatibility, availability, and tunability [26,27,28]. Chitosan has also demonstrated the ability to enhance immune responses following subcutaneous vaccinations, making it a promising vehicle for i.t. immunotherapy delivery [29,30].

Chitosan forms very strong inter- and intramolecular hydrogen bonds between its hydroxyl and amine groups. This gives chitosan its semicrystalline/crystalline structure [31]. Glycerol is a simple triol that is used as a plasticizer in biodiesel, bioproducts, and food production [32,33,34]. As a humectant with bacteriostatic properties, glycerol is also emerging as a popular component in wound healing biotechnology [35,36]. Glycerol has been shown capable of forming three hydrogen bonds with the glucosamine units of chitosan [37]. Because glycerol binds but does not crosslink chitosan, it increases the mobility of chitosan and disrupts its crystalline structure. As hydrogen bonds are readily disrupted under high-shear and reform under low-shear environments [38], hydrogels containing glycerol often display high elasticity or shear-thinning, self-healing properties, making them ideal for injection applications [39].

The current literature on chitosan–glycerol hydrogels for biomedical applications centers around dermal wound protection films [40,41,42]. While these works focus on the strong mechanical properties and porous structure advantages of this combination, limited exploration into injectable applications has been carried out [43]. Chitosan and β-glycerophosphate, a glycerol derivative with an attached phosphate group, have been extensively explored in drug delivery applications [44,45]. β-glycerophosphate is a phosphate donor which interacts electrostatically and crosslinks chitosan. In addition, the two remaining hydroxyl groups are able to form hydrogen bonds with chitosan’s amine and hydroxyl groups. Nevertheless, this combination relies on thermosensitive mechanisms requiring multiple minutes to fully achieve gelation. Prior to gelation, a significant fraction of the injectate may leak from injected tumors. As a result, shear-thinning, self-healing injectable hydrogels may be more effective than liquid-to-solid responsive hydrogels, although head-to-head comparisons are needed.

In this project, a chitosan–glycerol hydrogel was synthesized via neutralization and concentration of a viscous solution of chitosan and glycerol. Upon hydrogel formulation, gelation conditions were parameterized through the examination of factors including chitosan concentration, chitosan molecular weight (MW), degree of deacetylation (DDA), and chitosan:glycerol ratio. Rheology studies characterized the mechanical properties of the hydrogel. Cytotoxicity was evaluated via direct and indirect exposure of 3T3 fibroblasts to chitosan–glycerol hydrogels. The ability of chitosan–glycerol hydrogel to resist leakage and enhance the retention of co-formulated molecules was tested in tumor phantoms. Finally, in vitro release profiles were obtained with a range of fluorescent molecules to determine the performance of the hydrogel as a sustained release device.

## 2. Results and Discussion

### 2.1. A Novel Chitosan–Glycerol Hydrogel Is Created and Parameterized

**Figure 1:**
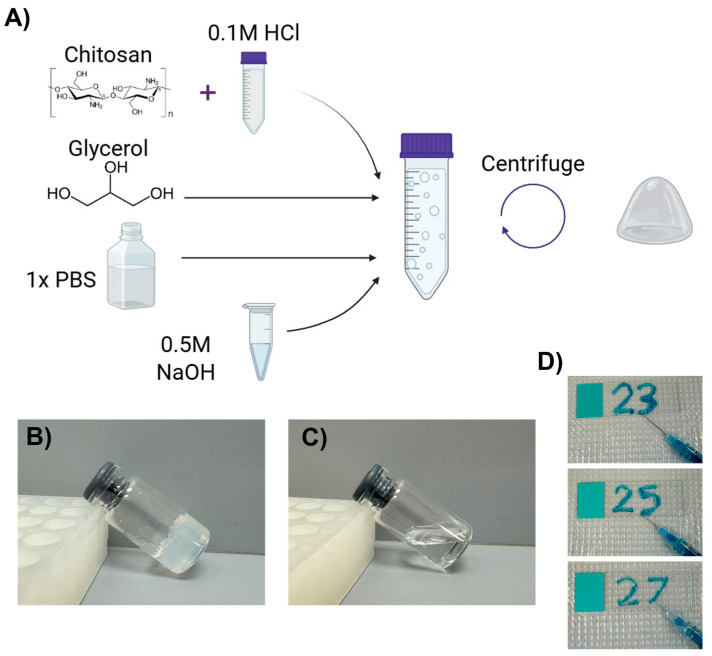
Chitosan–glycerol hydrogel creation and properties. (**A**) The synthesis scheme of chitosan–glycerol hydrogel includes solubilizing chitosan in HCl-acidified phosphate-buffered saline (PBS), adding glycerol, and then neutralizing with sodium hydroxide prior to centrifugation and inversion to remove excess liquid. Created with BioRender.com (May 2023). (**B**) Chitosan–glycerol hydrogel is non-flowable. (**C**) No hydrogel is formed without neutralization. (**D**) Chitosan–glycerol hydrogel was injectable through needles as small as 27-gauge. A small amount of methylene blue was added to the hydrogel for visualization purposes.

The co-formulation of glycerol with a chitosan solution in dilute hydrochloric acid led to hydrogel formation upon neutralization with sodium hydroxide and centrifugation to remove excess liquid. The general scheme to produce chitosan–glycerol hydrogel is shown in Figure 1A. While an intermediate precipitate formed upon neutralization, centrifugation of the combined solution yielded a solid hydrogel (Figure 1B). Similar results can be achieved using acetic acid in place of hydrochloric acid. Neutralization was found to be key as no gel was formed without this step (Figure 1C).

The hydrogel was determined to be a viscoelastic solid that recaptured its original shape after slight deformation and did not flow on a microscope slide. The hydrogel was injectable through needles as small as 27-gauge (Figure 1D). The pH of the hydrogel could be tuned within physiological range, 6.8 to 7.5, depending on relative amounts of acid and base.

After creating an initial hydrogel prototype, we wanted to understand the effects of different parameters such as chitosan concentration, DDA, MW, and chitosan:glycerol ratio on hydrogel formation. A broad range of starting materials were tested, and their resultant formulations were assessed by inspection. Formulations were characterized as a precipitate, a flowable liquid, or a non-flowable hydrogel.

**Figure 2:**
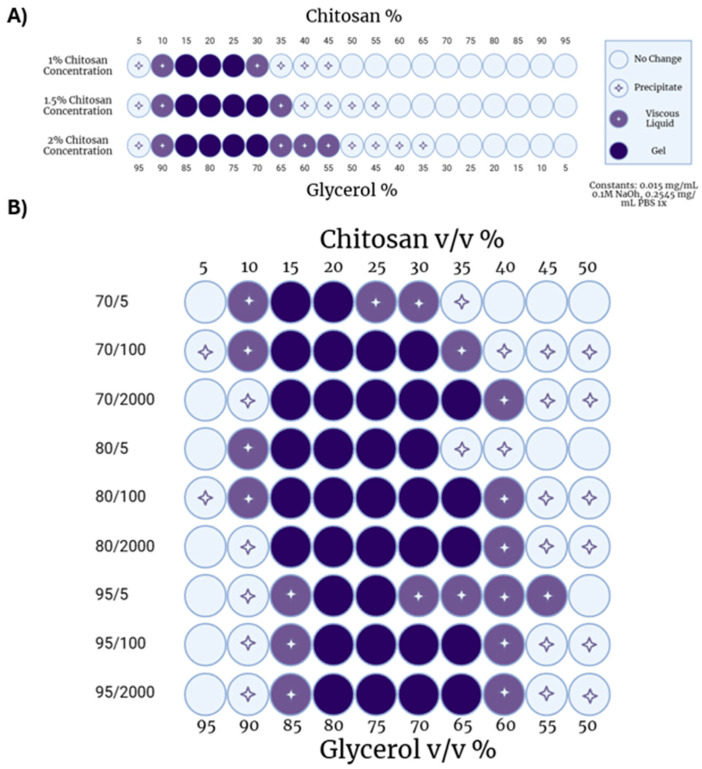
Chitosan–glycerol hydrogel parameterization. (**A**) Effect of chitosan solution concentration and chitosan:glycerol volume ratios on hydrogel gelation utilizing chitosan of average 70 DDA and 100 cP viscosity. (**B**) Effect of varied chitosan physiochemical factors on hydrogel gelation at various chitosan–glycerol relative volumes with constant 1.5% chitosan solution concentration. Created with BioRender.com (May 2023).

First, the effect of chitosan concentration on gelation was determined. Increasing the concentration of chitosan solution while keeping other parameters fixed modestly increased the number of conditions that led to hydrogel formation (Figure 2A). At 1% chitosan, gels were found when chitosan:glycerol volume ratios were between 15:85 and 25:75. At 1.5% and 2% chitosan, gels formed when chitosan:glycerol ratios were between 15:85 and 30:70. As chitosan concentration increased, the number of conditions producing viscous liquids also increased (Figure 2A).

Next, the MW and DDA of chitosan were varied at a fixed chitosan concentration (1.5% *w/v*) to determine the impact of chitosan’s physiochemical properties on hydrogel formation. DDA had a limited effect with 70%, 80% and 95% deacetylated chitosans resulting in similar hydrogel formations (Figure 2B). Upon closer inspection, 80% deacetylated chitosan formed hydrogels under a greater number of chitosan:glycerol ratios (14) compared to either 70% (11) or 95% (10) deacetylated chitosans. These data indicate that an intermediate level of deacetylation may be more forgiving for gelation.

Because chitosan is a polydisperse, natural polysaccharide, it does not exist at a single MW. Viscosity is often used as a surrogate for MW as it is easily measured and increases predictably with MW [46]. Thus, the viscosity in centipoise (cP) of a 1% chitosan in 1% acetic acid is reported as the number to the right of the ‘/’. Increasing viscosity reflects an increase in MW.

Studies exploring the effect of chitosan MW on hydrogel formation demonstrated that shorter-chain chitosans (70/5, 80/5, 95/5) formed fewer hydrogels (Figure 2B). This was expected as shorter chains have fewer opportunities to interact with each other. On the other hand, higher-MW chitosan with viscosities of 100 cP or 2000 cP, formed hydrogels when formulated with glycerol to the same relative extent. These data indicate that a threshold size of chitosan may be needed to maximize hydrogel formation.

To our knowledge, only one other publication similarly describes a hydrogel comprising only chitosan and glycerol with a neutralization-based gelation process [40]. Ramesan and Jain evaluated the potentiation of chitosan–glycerol hydrogel as a topical, protective barrier to prevent metal allergy [40]. The injectability, biocompatibility, and capabilities of retaining co-formulated therapeutics within chitosan–glycerol hydrogels were not previously explored.

Hydrogels created with chitosan and glycerol as the main reagents, along with additions such as dextran or fish gelatin, have found recent use in topical wound healing applications [47,48]. While these hydrogels are highly viscous and biocompatible, they do not display the injectability required for i.t. application. Kocak et al. discovered an injectable chitosan–glycerol–heparin hydrogel with a similar neutralization gelation mechanism for tissue regeneration applications [49]. However, the hydrogel was only injectable through needle gauges of 21 and larger, with most studies conducted with 18- and 19-gauge needles.

### 2.2. Chitosan–Glycerol Hydrogel Displays Shear-Thinning Behavior

**Figure 3:**
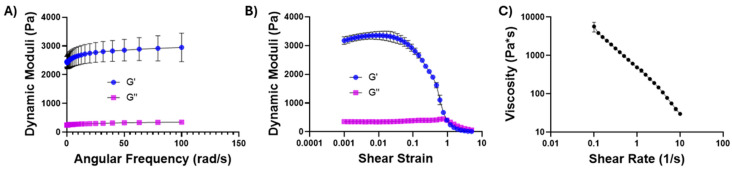
Rheological testing of chitosan–glycerol hydrogel. (**A**) Oscillatory frequency sweep performed at a strain of 1% (**B**); oscillatory amplitude sweep performed from shear strains of 0.1–500%; (**C**) viscosity sweep performed over shear rates of 0.1–10 Hz. Data are represented as the mean ± standard deviation for 3 replicates.

As indicated in Figure 1D, chitosan–glycerol hydrogel was injectable through thin needles and immediately reformed following injection, implying that it exhibited shear-thinning properties. Rheological testing was performed to confirm the unique mechanical properties of chitosan–glycerol hydrogel. Angular frequency sweeps confirmed the elastic solid nature of the chitosan–glycerol hydrogel through a storage modulus (G′) to loss modulus (G″) ratio greater than one for all frequency values (Figure 3A). Minor increases were seen in the storage modulus as frequency increased (10.88 ± 528% from 0.1 to 20 Hz and 17.47 ± 12.84% from 0.1 to 100 Hz), denoting a slightly stiffer nature as applied frequency increased. Amplitude sweeps show a small linear viscoelastic region (LVER) ending at a shear strain value of 0.0559, beyond which the storage modulus is generally dependent on the deformation force (Figure 3B). The negative slope of viscosity values as shear rate increases indicates shear-thinning behavior of the hydrogel, providing an explanation for the ease of injectability (Figure 3C). The chitosan–glycerol hydrogel demonstrated higher viscosity values at low shear rates compared than other literature-based chitosan hydrogel delivery systems [50,51,52], pointing to its strong potential as a viscoelastic injectable medium that is capable of resisting shear forces following i.t. injections.

### 2.3. Chitosan–Glycerol Hydrogel Resists Leakage from Injection Site

**Figure 4:**
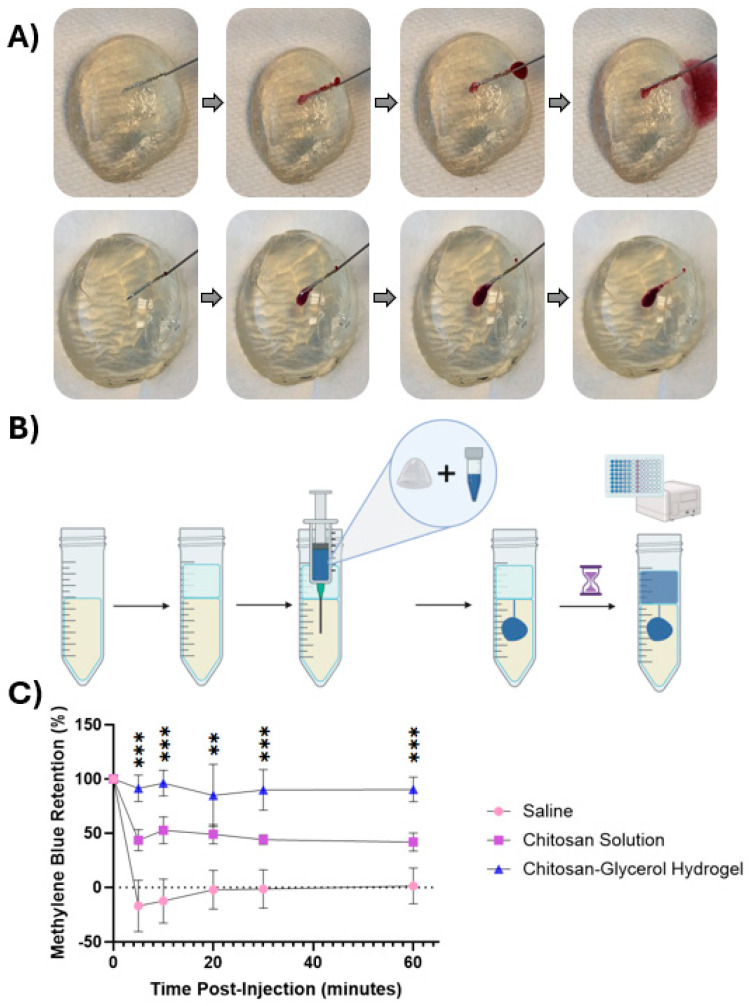
Injectability and retention of the chitosan–glycerol hydrogel in tumor phantoms. (**A**) Red dye formulated in saline solution (top row) rapidly exits the phantom along the needle track. In contrast, red dye formulated in chitosan–glycerol hydrogel (bottom row) is completely retained; (**B**) injection retention quantification protocol including the creation of an agar tumor phantom model and absorption reading of saline layer. Created with BioRender.com (May 2023) (**C**); retention values of a saline solution, 1.5% *w/v* chitosan solution, and chitosan–glycerol hydrogel loaded with 25 μg/mL methylene blue solution over 1 h. Data are represented as the mean ± standard deviation for 6 replicates. Mean comparison analysis between injection groups conducted from a one-way ANOVA (*n* = 3). ** *p* < 0.01, *** *p* < 0.001.

To directly visualize the potential of chitosan–glycerol hydrogel to resist short-term leakage after i.t. injection, optically clear tumor phantoms were injected with formulations containing dyes for visual or colorimetric analyses (Figure 4). Injections of saline containing red dye immediately leaked out of a gelatin tumor phantom along the needle track (Figure 4A, upper series). On the other hand, chitosan–glycerol hydrogels were completely retained within the tumor phantom (Figure 4A, lower series). An agar-based tumor phantom absorption experiment was then designed to quantify retention of various injectates (Figure 4B). As with the visual injection experiments, all of the saline-based injections leaked from the agar immediately (Figure 4C). Even at the first time point, which was 5 min post-injection, the mean retention of methylene blue (−16.92 ± 23.5%) was statistically indistinguishable from 0% retention. About half of the viscous chitosan solution leaked out of the phantom within 5 min, with only 43.6 ± 9.7% retained (Figure 4C). The remainder of the chitosan solution was retained for at least 1 h, indicating that viscous solutions can enhance local retention, albeit with significant immediate leakage of injectate. Injections of chitosan–glycerol hydrogel exhibited minimal leakage over the 1 h experiment (Figure 4C). There was no difference between the retention of chitosan–glycerol hydrogel at five minutes (91.4 ± 12.1%) and one hour (90.4 ± 11.4%). One-way ANOVA of mean retention percentages among the three groups was compared and found to be statistically different at each time point. While this trend will require in vivo validation, it provides promising evidence of the application potential of the chitosan–glycerol hydrogel for i.t. injection.

It should be noted that the immediate retention of injectable hydrogels provides a delivery advantage over the numerous responsive hydrogels currently under development [53,54]. Responsive hydrogels transition from injectable liquids into hydrogels upon a critical change in temperature, pH, or some other stimulus [55,56]. These transitions can take a few to several minutes, during which time the injected liquids are likely to be excluded from a tumor, as seen in our studies using saline or chitosan solution vehicles (Figure 4).

### 2.4. Chitosan–Glycerol Hydrogel Is Nontoxic

**Figure 5:**
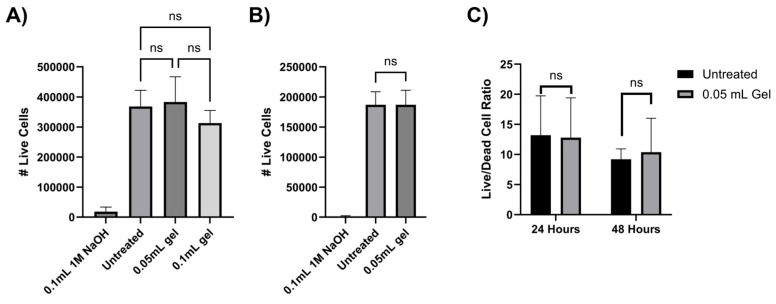
Cytotoxicity assessment of chitosan–glycerol hydrogel. (**A**) Indirect contact 24 h live cell counts obtained via trypan blue staining. Post hoc analysis conducted from a one-way ANOVA with Tukey’s test compares untreated and chitosan–glycerol-hydrogel-treated groups (*n* = 3). (**B**) Direct contact 24 h live cell fraction obtained via trypan blue staining normalized to untreated group. Two-tailed, unpaired t-tests were used for statistical comparison of untreated and chitosan–glycerol-hydrogel-treated groups (*n* = 8). (**C**) Indirect contact 48 h live/dead cell ratio obtained via trypan blue staining. Statistical analysis was conducted via two-tailed, unpaired t-test (*n* = 3). Data are represented as the mean + standard deviation ns: *p* > 0.05.

To evaluate the biocompatibility of chitosan–glycerol hydrogels, direct and indirect contact cell viability assays were performed in alignment with International Standard ISO 10993-1. For the indirect experiment, 3T3 murine fibroblasts seeded in the lower chamber of a transmembrane plate with chitosan–glycerol placed in the upper chamber. After 24 h, there were no significant differences in live cell counts between untreated and chitosan–glycerol-hydrogel-treated groups (Figure 5A). In the direct contact experiment, chitosan–glycerol hydrogel was mixed into the culture media before applying directly onto the fibroblasts. Once again, there were no significant differences in live cell counts when chitosan–glycerol hydrogel was added to the media (Figure 5B). To account for the possibility that the hydrogel increased cell death as well as proliferation, both live and dead cells were counted, and live/dead cell ratios were calculated over 48 h using the indirect setup. At both 24 and 48 h, there were no significant differences in live/dead cell ratios between untreated groups and cells exposed to chitosan–glycerol (Figure 5C). These data confirm that chitosan–glycerol does not impact cell viability.

Although glycerol can be toxic at high concentrations, it has been shown that glycerol binds strongly to chitosan and can be effectively immobilized [37]. As a result, despite the relatively high concentration of glycerol within the hydrogel, cells are not exposed to toxic levels.

### 2.5. Hydrogel Displays Varied Release Profiles by Molecular Weight and Saturation

**Figure 6:**
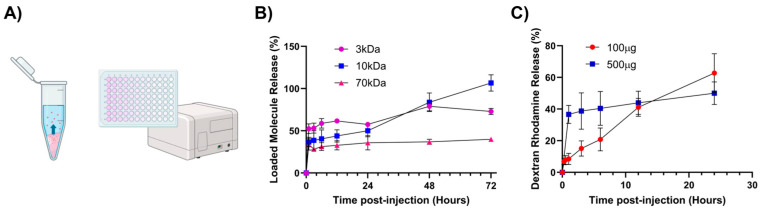
In vitro release of model therapeutics from chitosan–glycerol hydrogel. (**A**) Chitosan–glycerol hydrogels (100 μL) formulated with fluorescence labeled dextran were placed in microcentrifuge tubes and covered with 1 mL of PBS. The release of dextran into the PBS layer was quantified via fluorescence measurements. Created in BioRender.com (July 2024). (**B**) 72 h fluorescence release profiles of 3, 10, and 70 kDa molecules (500 μg in 100 μL hydrogel). (**C**) 24 h release profiles of 100 μg or 500 μg of 10 kDa Dextran, Rhodamine B loaded in chitosan–glycerol. Data are represented as the mean ± standard deviation.

In vitro release assays were performed to understand the release kinetics of macromolecules (Figure 6A). Fluoresence-labeled dextrans of different MWs were used to model a range of immunotherapeutics. Three kDa and 10 kDa dextrans were selected to represent small molecule immunotherapeutics such as peptides and agonists [57]. Seventy kDa dextran represents larger molecule immunotherapeutics such as multimeric cytokines and bispecific molecules [58,59,60].

All formulations exhibited a burst release over the first few hours (Figure 6B). In particular, a one-hour initial release burst was highest for 3 kDa dextran (52.5 ± 5.8%) followed by 10 kDa dextran (36.7 ± 5.7%) and 70 kDa dextran (34.0 ± 6.8%). After the initial burst, dextrans were released much more slowly. Only about 11.8 ± 1.0% of the 70 kDa dextran was released from 3 to 72 h. As expected, smaller dextrans were able to diffuse out of the hydrogel and into the saline much more quickly (Figure 6B). It should be noted that the 3 kDa dextran, which was fluorescein-labeled and thus negatively charged, released more slowly than 10 kDa tetramethylrhodamine-labeled dextran, which is slightly positive charged due to cationic tetramethyrhodamine. Since chitosan is polycationic, it is likely that negatively charged species interact electrostatically and hinder their release. Ongoing research is focused on elucidating the role of macromolecular charge on release from chitosan–glycerol gels.

In addition, we hypothesized that overloading the hydrogel may have contributed to the unexpected burst release. Thus, release profiles were generated at high and low dextran concentrations. Lower dextran loading (100 μg) showed a near-linear release over a 24 h time period. In contrast, the higher dextran concentration (500 μg) resulted in an initial burst followed by a slower, zero-order release profile (Figure 6C). These data suggest that dextran interacts with chitosan–glycerol hydrogel at low concentrations. However, at high concentrations, a significant fraction of dextran does not interact with the hydrogel and diffuses out of the hydrogel unhindered.

The effect of charge and size of a releasing therapeutic is the subject of future research. Given that dextran is a linear molecule, it has a higher probability of becoming entangled with hydrogel compared to the more globular configurations of most proteins. Nevertheless, data gathered in this project demonstrate that chitosan–glycerol hydrogels are simple, injectable vehicles for sustained, local delivery of macromolecular therapeutics.

## 3. Conclusions

To address issues with intratumoral injection retention, we developed and characterized a novel chitosan–glycerol hydrogel formulation. The hydrogel exhibits viscoelastic, mechanical, and is shear-thinning allowing for injection through needles as small as 27-gauge. Testing with cells and model therapeutics confirmed its nontoxicity and enhanced retention compared to less-viscous solutions. Finally, sustained release from the hydrogel was demonstrated through loading of model therapeutics of varying sizes. This promising blend of properties highlights the potential of the chitosan–glycerol hydrogel for future application in the intratumoral delivery of immunotherapeutics.

## 4. Materials and Methods

### 4.1. Materials and Reagents

Chitosan was sourced from Heppe Medical Chitosan (Halle, Germany). The following varieties of chitosan, listed as DDA/viscosity, were purchased: 70/10 (PN 24201), 70/100 (PN 24204), 70/1000 (PN 24207), 80/10 (PN 24401), 80/100 (PN 24404), 80/1000 (PN 24407), 95/10 (PN 24701), 95/100 (PN 24704), and 95/1000 (PN 24707)). Glycerol was purchased from Fisher BioReagents (Waltham, MA, USA) (BP229-1).

For tumor phantoms, Knox Unflavored Gelatine 16 oz and agar from ThermoFisher (Waltham, MA, USA) (A10752.22) were selected based on prior publications exploring in vitro tumor phantoms [61,62,63,64,65]. Fluorescence-labeled and dye molecules for release studies included methylene blue 1% from Biopharm Inc. (Hatfield, AR, USA) (BM8341), rhodamine b isothiocyanate dextran 10 kDa (Sigma-Aldrich R8881), dextran, fluorescein 3000 MW (D3305), 70,000 MW (D1823) and dextran, tetramethylrhodamine 10,000 MW (D1868) from ThermoFisher.

Murine NIH/3T3 fibroblasts originally isolated from a mouse NIH/Swiss embryo were purchased from the American Type Culture Collection (Manassas, VA, USA). Cells were cultured in DMEM (Corning (Corning, NY, USA), catalog no. 15-013-CV) with 1 mM penicillin-streptomycin (GenClone (El Cajon, CA, USA), catalog no.25-513), 10% fetal bovine serum (FBS) (GenClone, catalog no. 25-550), and 200 mM glutamine (Corning, catalog no. 25-005-CI) at 37 °C in 5% CO_2_ and 95% humidity.

### 4.2. Hydrogel Creation

A total of 300 mg of 70/100 chitosan was dissolved in 20 mL of 0.1 M hydrochloric acid (HCl) in a 50 mL conical tube rotating slowly overnight, to form a 1.5% (*w/v*) chitosan solution. The next day, 2.5 mL of this solution was then added to 7.5 mL of glycerol. After vortexing for 10 s, 3.5 mL of phosphate-buffered saline (PBS) was incorporated as a buffer. The solution was vortexed for another 10 s before 0.5 mL of 0.5 M sodium hydroxide (NaOH) was added to induce neutralization. The solution was then centrifuged at 10,000 rpm for 5 min. Excess liquid was then drained, and the viscous product was inverted for 24 h to support further drainage, yielding a chitosan–glycerol hydrogel. All steps were performed at room temperature.

### 4.3. Hydrogel Parameterization Testing

The chitosan–glycerol hydrogel creation process was repeated for chitosan solution concentrations of 1%, 1.5%, and 2% (*w/v*) chitosan in 0.1M HCl. Chitosan to glycerol relative volume ratios for 10 mL of total solution ranging from 5:95 to 95:5 by increments of +5/−5 were tested for each chitosan solution concentration. Degree of deacetylation (70, 80, 95) and viscosity (5, 100, 2000) combinations were also tested due to their status as the main biochemical differentiators between chitosan varieties [25].

### 4.4. Mechanical Property Characterization

Rheological testing was performed at room temperature using an Anton Paar Physica MCR 301 Rheometer with 500 μL of a 70/100, 1.5% *w/v* chitosan solution at a 1:3 ratio to glycerol. Oscillatory amplitude sweeps were performed from 0.1 to 500% shear strain at a frequency of 10 Hz, and oscillatory frequency sweeps were performed from 0.1 to 100 Hz at a strain of 1%. Viscosity measurements were then obtained through a rotational continuous flow experiment over shear rates of 0.1–10 Hz to demonstrate shear-thinning.

### 4.5. Injection Retention Testing

Gelatin tumor phantoms for qualitative retention testing were formed by dissolving gelatin powder in deionized water at a concentration of 8.5 g/L at 80° C for 10 min under constant stirring in 100–500 mL beakers. The gelatin solution was then set at room temperature in half-sphere molds. Tumor phantoms were injected with approximately 100 μL of red food color dye formulated in either saline or chitosan–glycerol hydrogel. Images were acquired during injection, at the end of injection, and when the needle was fully removed from the injection track. Agar tumor phantoms for quantitative retention testing were formed by dissolving agar powder in deionized water at a concentration of 25 g/L at 100 °C under continuous stirring in a 500 mL beaker. 30 mL of agar solution were poured into a 50 mL centrifuge tube and allowed to solidify at room temperature. A 10 mL layer of PBS was added on top of the solidified agar. Nine 30 mL tumor phantoms were created in total. Three rounds of injections were prepared for addition into the agar tumor phantoms: a PBS solution, a 1.5% *w/v* chitosan solution in 0.1M HCl, and the chitosan–glycerol hydrogel formed with a 1.5% *w/v* 70/100 chitosan solution and a 1:3 chitosan:glycerol relative volume ratio. Methylene blue was added to 100 μL of each delivery medium at a concentration of 25 μL/mL and injected into individual tumor phantoms at depth of 10 mL into the agar layer. Absorbance readings were then taken at wavelengths of 655 nm and 670 nm at time points of 5, 10, 20, 30, and 60 min with 100 μL samples from the saline layer. Values were compared to absorbance readings at the same wavelengths of PBS and 100 μL of 25 μL/mL methylene blue in PBS diluted into 10 mL of PBS. All retention studies were performed at room temperature.

### 4.6. Cell Viability for Biocompatibility

For indirect cytoxicity studies, the bottom chambers of 24-well transmembrane plates were filled with 0.5 mL of DMEM growth media containing 200,000 3T3 cells. After 12 h incubation, cells were then exposed to experimental groups of 0 mL, 0.05 mL, and 0.1 mL of chitosan–glycerol hydrogel loaded onto upper chamber membranes and submerged in cell media. Control groups included 0.1 mL 1M NaOH and no treatment. After 24 h of exposure, membranes were removed, and cells were harvested and counted manually using a hemocytometer with trypan blue staining. A 48 h indirect contact cytotoxicity test was also performed to compare live: dead cell ratio of an untreated group with an experimental group exposed to 0.05 mL of chitosan–glycerol hydrogel via transmembrane well at 24 and 48 h time points. For direct contact cytotoxicity studies, 24-well plates were seeded with 0.5 mL of growth media containing 400,000 3T3 cells for 12 h. Media was then aspirated and replaced with 0.5 mL of fresh growth media containing 0.1 mL of chitosan–glycerol hydrogel. Live cells were counted 24 h after the incorporation of the hydrogel media utilizing trypan blue staining.

### 4.7. Release Profiles

Ten μL of 10 mg/mL fluorescein-labeled dextran (MW 3 kDa and 7 kDa) or tetramethylrhodamine-labeled dextran (MW 10 kDa) in deionized water (diH_2_O) was loaded in 100 μL of 70/100, 1.5% *w/v* chitosan–glycerol gel with a 1:3 chitosan:glycerol ratio via vortexing for one minute. This process was repeated in triplicate for each of the tested molecular weights. A total of 1 mL of PBS was then carefully added on top of each chitosan–glycerol hydrogel within microcentrifuge tubes. At time points of 1, 3, 6, 12, 24, 48, and 72 h, 50 μL samples were taken from the PBS layer and further diluted with 150 μL of PBS in microcentrifuge tubes before being covered in foil and frozen at −20 °C. After the conclusion of all time points, the resultant samples were thawed to room temperature and placed in a 96-well fluorescence microplate. Experimentation was repeated for 10 μL loading volume. Fluorescence concentration values were then obtained on a Biotek Cytation 5 plate reader with extended gain, 100 ms of delay, 10 measurements per data point, and a read height of 7 mm. Excitation and emission values were set to 490/20 and 525/20, respectively, for dextran fluorescein and 555/20 and 580/20, respectively, for dextran tetramethylrhodamine. Fluorescence standards included unmodified PBS 1X (0%) and PBS at the maximum potential release concentration, calculated as loaded dextran mass divided by saline layer volume. All release studies were conducted at room temperature.

## Data Availability

The original contributions presented in this study are included in the article. Further inquiries can be directed to the corresponding author.
